# Efficacy of metformin therapy in patients with cancer: a meta-analysis of 22 randomised controlled trials

**DOI:** 10.1186/s12916-022-02599-4

**Published:** 2022-10-24

**Authors:** Jie Wen, Zhenjie Yi, Yuyao Chen, Jing Huang, Xueyi Mao, Liyang Zhang, Yu Zeng, Quan Cheng, Wenrui Ye, Zhixiong Liu, Fangkun Liu, Jingfang Liu

**Affiliations:** 1 Department of Neurosurgery, Xiangya Hospital, Central South University, Changsha, Hunan China; 2 Hypothalamic Pituitary Research Center, Xiangya Hospital, Central South University, Changsha, Hunan China; 3 Department of Epidemiology and Health Statistics, Xiangya School of Public Health, Central South University, Changsha, Hunan China; 4 National Clinical Research Center for Mental Disorders and Department of Psychiatry, The Second Xiangya Hospital of Central South University, Changsha, Hunan China; 5 National Clinical Research Center for Geriatric Disorders, Xiangya Hospital, Central South University, Changsha, Hunan China

**Keywords:** Metformin, Cancer, RCTs, Meta-analysis, Cancer-related mortality

## Abstract

**Background:**

To investigate whether metformin monotherapy or adjunctive therapy improves the prognosis in patients with any type of cancer compared to non-metformin users (age ≥18).

**Methods:**

Databases (Medline, Embase, and the Cochrane Central Register of Controlled Trials) and clinical trial registries (ClinicalTrials.gov; the World Health Organization International Clinical Trials Registry Platform) were screened for randomized, controlled trials (RCT) reporting at least progression-free survival (PFS) and/or overall survival (OS). Main outcome measures included hazard ratios (HR), and combined HRs and 95% confidence intervals (CI) were calculated using random-effects models.

**Results:**

Of the 8419 records screened, 22 RCTs comprising 5943 participants were included. Pooled HRs were not statistically significant in both PFS (HR 0.97, 95% CI 0.82–1.15, *I*^*2*^ = 50%) and OS (HR 0.98, 95% CI 0.86–1.13, *I*^*2*^ = 33%) for patients with cancer between the metformin and control groups. Subgroup analyses demonstrated that metformin treatment was associated with a marginally significant improvement in PFS in reproductive system cancers (HR 0.86, 95% CI 0.74–1.00) and a significantly worse PFS in digestive system cancers (HR 1.45, 95% CI 1.03–2.04). The PFS or OS was observed consistently across maintenance dose, diabetes exclusion, median follow-up, risk of bias, and combined antitumoral therapies.

**Conclusion:**

Metformin treatment was not associated with cancer-related mortality in adults compared with placebo or no treatment. However, metformin implied beneficial effects in the PFS of the patients with reproductive system cancers but was related to a worse PFS in digestive system cancers.

**Systematic review registration:**

PROSPERO registration number CRD42022324672.

**Supplementary Information:**

The online version contains supplementary material available at 10.1186/s12916-022-02599-4.

## Background

Cancer death accounts for 21% of all cases in both men and women in the USA, and cancer is the second leading cause of death worldwide [1]. Of all incident cases, lung and bronchus cancer, prostate cancer, and colorectal cancer (CRC) account for the largest percentages in men. New diagnoses for women mostly include breast cancer (BC), lung cancer, and CRC. The statistics in 2020 showed that the risk of cancer death was accumulating regardless of the social development level [2]. Moreover, it is estimated that 19.3 million new cancer cases and almost 10.0 million deaths from cancer will occur in 2020.

Metformin is the first-line drug for type 2 diabetes (T2D) patients, which induces a hypoglycemic effect by targeting and activating the enzyme AMP-activated protein kinase (AMPK) and inhibiting hepatic glucose production. The activation of the AMPK-pathway may reduce the activity of insulin in promoting tumor progression and can inhibit the mammalian target of rapamycin (mTOR), which is closely connected to tumor cell proliferation [3–6]. In 2005, Evans et al. [7] retrospectively identified that metformin is related to a lower risk of developing cancer in patients with T2D, generating considerable publicity over the anticancer effect of metformin. In recent years, metformin has been advocated as a potential economic strategy to improve the prognosis in both diabetic and nondiabetic cancer patients.

However, the available results are controversial. Several studies and meta-analyses have indicated that metformin therapy is associated with reduced cancer-related mortality [8–12], while others point out that concomitant medication with metformin showed no significant effect on cancer-related mortality [13–18] or even led to inferior outcomes [19]. In the last decade, several randomized controlled trials (RCTs) have been conducted to assess the effectiveness of metformin monotherapy or adjunctive therapy in antitumor medications. We carried out a meta-analysis of RCTs to evaluate whether metformin reduces cancer-related mortality in adults compared with placebo or no treatment.

## Methods

This prospective study was performed following the Preferred Reporting Items for Systematic Reviews and Meta-Analysis (PRISMA) [20]. The protocol was registered with PROSPERO (CRD42022324672).

### Eligibility criteria

The inclusion and exclusion criteria were prespecified. The inclusion criteria contained several essential factors, including (1) RCTs if metformin was one of the randomized therapies; (2) investigation of the efficacy of metformin monotherapy or as an adjunctive therapy comparing the treatment group with a control group (placebo or no treatment); (3) investigation of adults (age ≥ 18 years) with any type of cancer; and (4) presence of reported results on progression-free survival (PFS) and/or overall survival (OS). If the studies did not report PFS or OS, we contacted the investigators by e-mail, requesting them to provide survival data. Studies were excluded if they (1) were case reports, retrospective studies, observational studies, or post hoc analyses of RCTs; (2) synchronously used other antidiabetic drugs; or (3) had no available results related to survival.

### Search strategy

Electronic searches of databases (Medline, Embase, and the Cochrane Central Register of Controlled Trials) and clinical trial registries (ClinicalTrials.gov; the World Health Organization International Clinical Trials Registry Platform) were conducted from their inception to June 1, 2022. To maximize the search for relevant trials, we hand-searched the bibliographies of identified studies and systematic reviews. Language restrictions were not applied to the search. Additional file 1 shows the detailed search strategy.

### Study selection

All retrieved studies were screened by two independent researchers (ZY and JW) for titles and abstracts to evaluate their eligibility. Full-text publications or presentations were retrieved for further assessment when the information was insufficient. When studies had multiple publications or overlapping patients, the most recent publication was chosen.

### Data collection

Data on the study designs, patient characteristics, interventions, and outcomes were collected from the included studies into a standard sheet by two independent researchers (JW and YZ). The hazard ratios (HR) included associated data that were either directly collected from the studies or assessed from Kaplan–Meier curves [21]. The adjusted HRs were extracted in preference to unadjusted HRs if provided by the studies.

### Risk of bias assessment

The risk of bias in each trial was evaluated using the Cochrane Risk of Bias Assessment Tool (version 2) [22]. We scored every trial as low risk, with some concerns, or high risk based on the following criteria: (1) randomization process, (2) deviations from intended interventions, (3) missing outcome data, (4) measurement of the outcome, and (5) selection of the reported result [22]. Two researchers (JW and FL) independently assessed the potential study bias of the included studies. Disagreements were resolved by consensus.

### Subgroup analyses

We performed several subgroup analyses to evaluate the interactions according to the maintenance dose ([500, 1000), [1000, 1500), [1500, 2000), [2000, 2500) mg), diabetes exclusion (yes or no), risk of bias (low risk, some concerns, high risk), and combination with chemotherapy, radiotherapy (yes or no), and targeted therapy (yes or no). Previous studies have shown that cancers within the same system owned similar molecular characteristics [23–25]; therefore, we conducted retrospective subgroup analyses of the cancer type based on the systems that they originated from (reproductive, respiratory, or digestive system cancers).

### Statistical analysis

The primary endpoints were the PFS and OS of cancer patients, measured by HRs. We performed statistical analyses based on the intention-to-treat results using the meta package in R (version 4.1.3). HRs and their 95% confidence intervals (CI) were used to assess outcomes, and *P* < 0.05 was considered statistically significant. Heterogeneity was estimated with the *I*^*2*^ test [26]. The assumption of heterogeneity was deemed valid for *I*^2^ >  25% and *P* < 0.10 as in a previous study [27]. If heterogeneity was not significant, we used fixed-effects models to pool outcomes. When heterogeneity was significant, we used random-effects models. Meta-regression and sensitivity analyses were performed to investigate potential sources of heterogeneity. Qualitative and quantitative assessments of small-study effects were performed with the funnel plot and Egger’s test [28, 29].

## Results

### Eligible studies and study characteristics

**Fig. 1:**
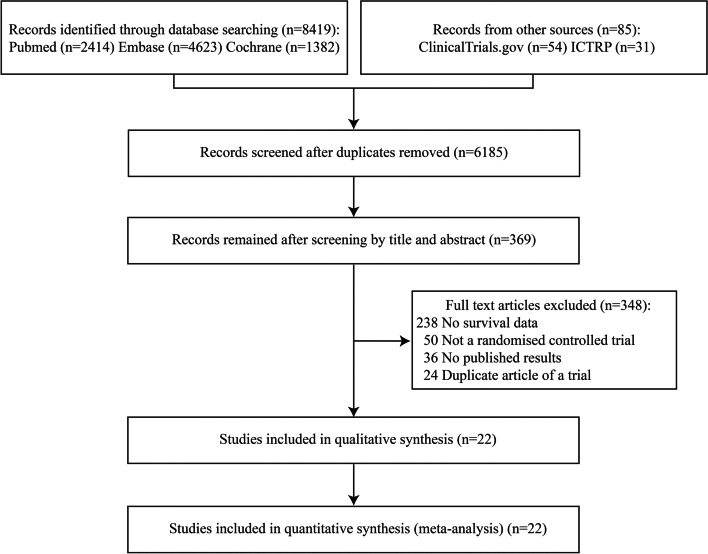
Search and selection of eligible studies for inclusion

We screened 8419 records and identified 22 eligible trials (5943 participants) in the final meta-analysis (Fig. 1) [30–52]. All the studies were RCTs published between 2015 and 2022. The number of recruited participants in the included trials ranged from 25 to 3649. The mean age of the metformin and control groups was 58.6 and 58.9 years, respectively. The female proportion in the metformin and control groups was 64% and 65%, respectively. The population characteristics of the included trials are summarized in Additional file 2.

All eligible studies comprised patients with reproductive (breast, ovary, endometrium, and prostate), respiratory (lung), and digestive (pancreas and liver) system cancers. Seventeen of the 22 studies were performed on those with advanced or metastatic cancer. All studies administered antitumor therapies to the patients, including chemotherapy, radiotherapy, targeted therapy, hormone therapy, and immunotherapy. Fifteen studies excluded patients with diabetes at the inclusion stage of the trials. Six studies included patients with and without diabetes and one included only patients with metabolic syndrome. A diagnosis of diabetes was noted in 310 (5%) of 5943 patients. All studies reported daily maintenance doses of metformin ranging from 500 to 2000 mg. All studies controlled or evaluated potential confounders; they conducted stratified randomization, reported balanced confounding factors in the metformin and control groups, or adjusted HRs by multivariable analysis. Table 1 summarizes the main characteristics of the studies. The methodological quality of the eligible studies was generally moderate to good (shown in Additional file 3: Figs. S1 and S2). The main source of bias was a lack of reporting if the allocation sequence was concealed until enrollment and assignment.

**Table 1:** Main characteristics of included studies

**Author **	**Year**	**Country**	**Study design**	**Cancer location**	**Stage/other restriction**	**Combined therapy**	**DM status** **(DM/non-DM)**	**Sample size** **(metformin/control)**	**Maintenance dose**	**Median follow-up** **(months)**	**Outcome type**	**Potential confounders control**			**Risk of bias**
												**S**^a^	**R**^a^	**M**^c^	
Goodwin	2022	Multiple centers	Double-blinded	Breast	High-risk nonmetastatic	Chemotherapy + radiotherapy + hormone therapy + targeted therapy^d^	No	3649 (1824/1825)	850mg bid	96.2 for ER/PgR+94.1 for ER/PgR-	OS	√	√	√	Low risk
Pimentel	2019	Canada	Double-blinded	Breast	Metastatic or unrectable locally advanced	Chemotherapy + hormone therapy	No	40 (22/18)	850mg bid	Not given	PFS, OS	√	√	√	Low risk
Nanni	2019	Italy	Open-label	Breast	Stage IV/ metastatic, HER2-negative	Chemotherapy	No	122 (57/65)	1000mg bid	39.6	PFS, OS	√	√	√	Some concerns
Zhao	2017	China	Open-label	Breast	Metastatic or locally advanced	Hormone therapy	No	60 (30/30)	500mg bid	22.3	PFS, OS	×	√	×	Some concerns
Salah	2021	Egypt	Open-label	Breast	Stage IV/ metastatic	Chemotherapy	No	50(25/25)	1000mg bid	6	PFS, OS	×	√	×	Some concerns
Liubota	2018	Ukraine	Not given	Breast	Stage II-III	Chemotherapy + hormone therapy	Yes	72(36/36)	500mg tid	39	PFS, OS	×	√	×	Some concerns
EL-Haggar	2016	Egypt	Not given	Breast	Newly diagnosed	Chemotherapy + hormone therapy	No	102(51/51)	850mg bid	Not given	PFS	×	√	√	Some concerns
Hamedi	2018	Iran	Open-label	Ovary	Epithelial	Chemotherapy	No	70 (30/40)	500mg tid	Not given	PFS	×	√	×	Low risk
Zheng	2019	China	Open-label	Ovary	Epithelial	Chemotherapy	No	44 (20/24)	850mg qd	Not given	PFS	×	√	×	Some concerns
Bae-Jump	2020	USA	Double-blinded	Endometrium	Stage III-IV/ recurrent	Chemotherapy	Mixed (102/367)	469 (234/235)	850mg bid	28	PFS, OS	√	√	√	Some concerns
Alghandour	2021	Egypt	Double-blinded	Prostate	High localized or node invasion or metastatic hormone sensitive	Chemotherapy + radiotherapy + hormone therapy	Mixed (15/99)	124 (62/62)	850mg bid	22	PFS, OS	√	√	√	Low risk
Martin	2021	France	Double-blinded	Prostate	Metastatic or hormone resistant	Chemotherapy	No	99 (50/49)	850mg bid	86	PFS, OS	×	√	×	Some concerns
Li	2019	China	Double-blinded	Lung	Stage IIIB-IV/ EGFR mutated NSCLC	Targeted therapy	No	202 (97/105)	1000mg bid	19.15	PFS, OS	×	√	×	Low risk
Arrieta	2019	Mexico	Open-label	Lung	Stage IIIB-IV/ EGFR mutated lung adenocarcinoma	Targeted therapy	No	139 (69/70)	500mg bid	16.9	PFS, OS	×	√	√	Some concerns
Lee	2021	South Korea	Open-label	Lung	Stage IIIB-IV/ EGFR-ALK wild NSCLC	Chemotherapy	Mixed (36/129)	165(82/83)	1000mg bid	32.4	PFS, OS	√	√	√	Some concerns
Marrone	2018	USA	Open-label	Lung	Stage IIIB-IV/ nonsquamous NSCLC	Chemotherapy + targeted therapy	No	24 (18/6)	1000mg bid	Not given	PFS, OS	×	√	×	Some concerns
Sayed	2015	Egypt	Open-label	Lung	Stage IV/ NSCLC	Chemotherapy	No	30 (15/15)	500mg qd	Not given	OS	√	√	×	Low risk
Skinner	2021	USA	Open-label	Lung	Stage III/ unresectable NSCLC	Chemotherapy + radiotherapy	No	167 (86/81)	1000mg bid	27.7	PFS, OS	√	√	√	Some concerns
Tsakiridis	2021	Canada	Open-label	Lung	Stage III/ Unresected locally advanced NSCLC	Chemotherapy + radiotherapy + immunotherapy^e^	No	54 (26/28)	1000mg bid	Not given	PFS, OS	√	√	√	Low risk
Kordes	2015	Netherlands	Double-blinded	Pancreas	Metastatic or unresectable locally advanced	Chemotherapy + targeted therapy	Mixed (14/107)	121 (60/61)	1000mg bid	28.1	PFS, OS	√	√	√	Low risk
Reni	2016	Italy	Open-label	Pancreas	Metastatic	Chemotherapy	Mixed (23/37)	60 (31/29)	2000mg daily	Not given	PFS, OS	×	√	√	Some concerns
Shorbagy	2020	Egypt	Not given	Liver	Advanced	Targeted therapy	Mixed (48/32)	80 (40/40)	500mg bid	Not given	PFS, OS	×	√	×	Some concerns

*ER/PgR* estrogen receptor and/or progesterone receptor, *HER* human epidermal growth factor receptor 2, *PFS* progression-free survival, *OS* overall survival, *NSCLC* non-small-cell lung cancer

^a^S=Stratified randomization

^a^R=Reported and no significant difference between metformin and control group

^c^M=Conducted multivariate analysis

^d^74.1%, 88.9%, 61.4%, 17.2% patients taking radiotherapy, chemotherapy, hormone therapy, targeted therapy, respectively

^e^20.3% patients taking immunotherapy

### Efficacy of metformin in patients with cancers

**Fig. 2:**
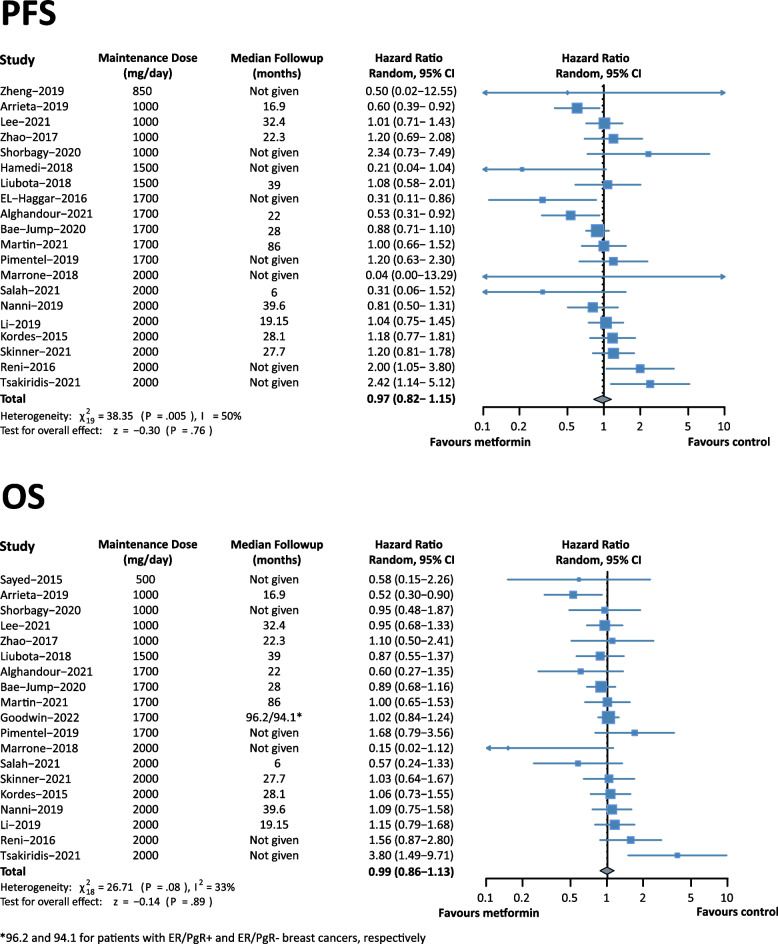
Forest plot of PFS and OS of trials evaluating metformin use

All 22 trials reported survival data, of which 20 and 18 reported PFS and OS, respectively. Both PFS (HR 0.97, 95% CI 0.82–1.15, *I*^2^ = 50%) and OS (HR 0.99, 95% CI 0.86–1.13, *I*^2^ = 33%) showed no significant difference between the metformin and control groups for patients with cancers (Fig. 2). Due to the heterogeneity, we applied a random-effects model to pool the HRs results. Our sensitivity analyses revealed that excluding any single study did not significantly affect the pooled estimate (Additional file 3: Figs. S3 and S4).

**Fig. 3:**
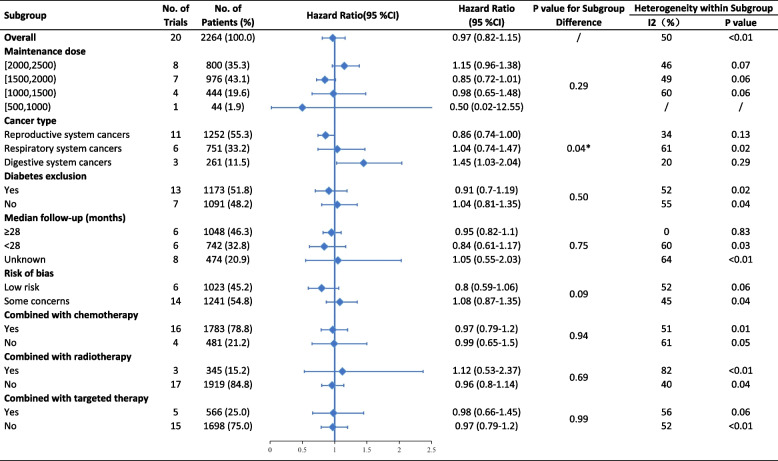
Subgroup analyses for PFS

**Fig. 4:**
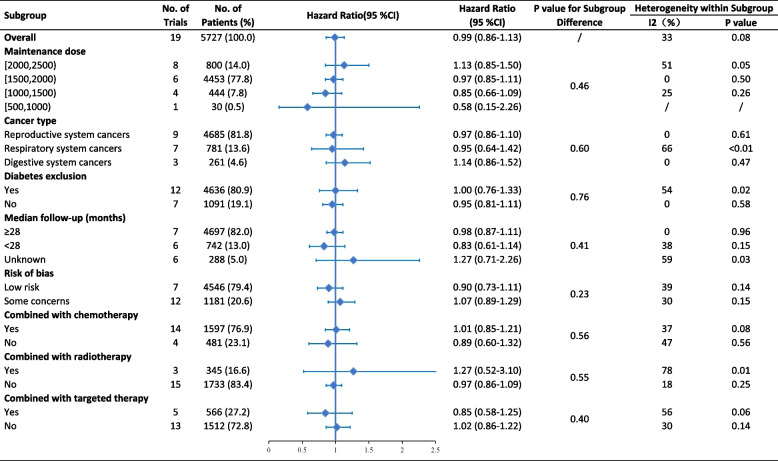
Subgroup analyses for OS

Subgroup analyses indicated that metformin use resulted in marginally significant improvement in PFS for patients with reproductive system cancers (HR 0.86, 95% CI 0.74–1.00). For digestive system cancers, metformin use showed significantly worse PFS (HR 1.45, 95% CI 1.03–2.04) (Fig. 3). The difference between subgroups based on cancer type was statistically significant in PFS (*p* = 0.04) but not in OS (*p* = 0.60) (Fig. 4). There was no clear evidence of between-subgroup differences based on maintenance dose, diabetes exclusion, median follow-up, risk of bias, and combined antitumoral therapies, neither in PFS nor in OS. Meta-regression revealed that the maintenance dose is not significantly correlated with improved OS (*p* = 0.07, coefficient = 0.0003, Additional file 3: Fig. S5). The subgroups’ meta-regression of the maintenance dose for PFS (*p* = 0.38) and median follow-up revealed no significant differences (*p* = 0.45 for PFS, *p* = 0.32 for OS).

The funnel plot analysis did not show substantial asymmetry (Additional file 3: Fig. S6). We did not observe evidence of small-study effects, with Egger *p* values of 0.58 for PFS and 0.66 for OS.

## Discussion

With individual participant data from 22 high-quality randomized controlled trials for more than 5943 patients with cancer, our meta-analysis revealed that metformin treatment was not associated with cancer-related mortality in adults compared with placebo or no treatment. Subgroup analysis suggests that metformin therapy is potentially beneficial for reproductive system cancers, including breast, ovary, endometrium, and prostate, but may be related to a worse prognosis for digestive system cancers, including pancreas and liver.

The effect of metformin in the prevention of reproductive system cancer progression may be related to its impact on the gonadal hormone levels. Metformin was reported to be effective in preventing hormone-related tumor progression, including breast [53], prostate, ovarian, and endometrial [54] cancers. Previous studies have reported that progestin can activate the PI3K/Akt pathway without progesterone receptor (PgR) mediation [55], and metformin suppresses both estrogen receptor (ER)/PgR signaling and PI3K/AKT/mTOR signaling to inhibit estradiol and progesterone-associated abnormal cell proliferation and hormone therapy resistance [56–58]. Recently, the largest RCT (MA.32), which enrolled 3649 patients with early BC, suggested prognostic benefits of metformin among HER2 + subtypes [39]. The addition of metformin did not reveal significant improvement in the total study population. However, the trial used invasive disease-free survival (IDFS) as a primary outcome instead of PFS, which placed more emphasis on cancer invasiveness. In addition, metformin exposure can affect human and mouse fetal testicular cells, thus reducing the production of androgens and testosterone [59]. Androgen signaling directly regulates Tcf7 and induces CD8+ T cell depletion, and higher mortality in men is observed with the development and progression of tumors in various organs [60]. A recent randomized trial of metformin treatment for 1 month found significantly lower testosterone concentrations in T2D men regardless of changes in blood glucose and weight [61]. For prostate cancer, androgen deprivation therapy (ADT) alone remains the first-line treatment in most cases. Pre-surgical administration of metformin in prostate cancer reduced the Ki-67 proliferation index by 29% compared with pretreatment biopsy [62]. A similar effect of metformin pre-surgical treatment in reducing tumor Ki-67 expression was also reported in endometrial cancer [63]. Further, metformin treatment was shown to reverse endometrial hyperplasia in a rat model [63, 64] and women with polycystic ovary syndrome (PCOS) [65], indicating metformin’s potential role in cancer prevention. Metformin has also shown anticancer effects in human ovarian cancer cells through ASK1-mediated mitochondrial damage and ER stress [66]. Our results concur with the findings of previous studies and support the more in-depth clinical investigations of the effect of metformin on hormone-related cancers.

Metformin monotherapy or combination therapy is associated with a worse prognosis in digestive system cancers, including pancreatic and liver cancers. Evidence from retrospective studies also indicates that chronic metformin treatment is related to enhanced tumor aggressiveness and sorafenib resistance in hepatocellular carcinoma [67, 68]. In two metastatic and advanced pancreatic cancer cohorts, the increased toxic effects of metformin were observed, such as esophagitis and lung infections, which limited their tolerance to originally prescribed doses of chemoradiotherapy and worsened the prognosis.

A significant association in the meta-regression between a low maintenance dose and prolonged OS was identified in our results. One possible explanation is that metformin may induce biphasic actions in various cell types, mostly showing a desirable effect at low concentrations and an undesirable or even toxic effect at high concentrations [69–81]. Furthermore, adverse effects of metformin, particularly diarrhea, have been reported to be dose-dependent [82, 83], may influence medication adherence and lead to poor treatment effects.

## Limitations

Our findings are based on large samples from high-quality RCTs with relatively long-term follow-up and with between-study heterogeneity as low or medium, indicating that our conclusions are relatively reliable. However, there are several significant limitations. First, there was evidence indicating that the effect of metformin use on the survival of patients with diabetes depends on the cumulative metformin dose [84, 85]. However, we could not obtain baseline cumulative dose values and the duration of medication for each individual; therefore, we were unable to analyze the effects of cumulative metformin dose on PFS and OS. Future studies should pay more attention to the effect of the cumulative metformin dose on the survival of cancer patients. Second, as cancer treatment has entered the epoch of precision medicine, the number of included studies was limited, and more research is required to further classify cancers, such as classification on the organ level and even pathological diagnoses on the molecular level.

## Conclusions

Metformin treatment was not associated with cancer-related mortality in adults compared with placebo or no treatment. However, metformin showed potentially beneficial effects on the PFS of the patients with reproductive system cancers but was related to a worse PFS in patients with digestive system cancers. The positive or desired effects may be maximal in low-dose conditions. Further studies are required to elucidate the effects and underlying mechanisms in specific cancer subtypes.

## Supplementary Information

**Additional file 1: Table S1.** Search strategy.

**Additional file 2: Table S1.** Population characteristics of included studies.

**Additional file 3: Fig. S1.** Risk of bias summary. **Fig. S2.** Risk of bias graph for each included study. **Fig. S3.** Sensitivity analyses for PFS. **Fig. S4.** Sensitivity analyses for OS. **Fig. S5.** Meta-regression for OS by maintenance dose. **Fig. S6.** Funnel plots for PFS and OS.

## Data Availability

All data generated or analyzed during this study are available in the article, additional files, or from the corresponding author upon reasonable request. The study protocol can be accessed on PROSPERO (CRD42022324672).

## Abbreviations

RCT: Randomized controlled trials
PFS: Progression-free survival
OS: Overall survival
HR: Hazard ratios
CI: Confidence interval
BC: Breast cancer
CRC: Colorectal cancer
T2D: Type 2 diabetes
AMPK: AMP-activated protein kinase
mTOR: Mammalian target of rapamycin
PgR: Progesterone receptor
ER: Estrogen receptor
