# Supplementary material for: Efficacy of metformin therapy in patients with cancer: a meta-analysis of 22 randomised controlled trials
Source: BMC Med. 2022 Oct 24;20:402. doi: 10.1186/s12916-022-02599-4 (PMC9594974; doi:10.1186/s12916-022-02599-4)
Supplement: Supplementary file 1 — Additional file 1: Table S1. Search strategy. [file 12916_2022_2599_MOESM1_ESM.docx]

**Additional file 1**

**Table S1**: Search strategy

| Pubmed | | |
| --- | --- | --- |
| #1 | neoplasms[MeSH Terms] | 3690422 |
| #2 | (tumor[Title/Abstract]) OR (tumo*[Title/Abstract]) OR (neoplasm[Title/Abstract]) OR (neoplas*[Title/Abstract]) OR (cancer[Title/Abstract]) OR (cancer*[Title/Abstract]) OR (malignan*[Title/Abstract]) OR (benign[Title/Abstract]) OR (carcinoma[Title/Abstract]) OR (carcinoma*[Title/Abstract]) | 3840793 |
| #3 | #1 OR #2 | 4866966 |
| #4 | metformin[MeSH Terms] | 16439 |
| #5 | biguanide[MeSH Terms]­ | 30829 |
| #6 | (metformin[Title/Abstract]) OR (dimethylbiguanidine[Title/Abstract]) OR (dimethylguanylguanidine[Title/Abstract]) OR (glucophage[Title/Abstract]) OR (biguanide[Title/Abstract]) OR (biguanid*[Title/Abstract]) | 27403 |
| #7 | #4 OR #5 OR #6 | 42216 |
| #8 | randomized controlled trials as topic[MeSH Terms] | 159302 |
| #9 | controlled clinical trials as topic[MeSH Terms] | 164981 |
| #10 | (randomized controlled trial[Title/Abstract]) OR (randomized controlled trials[Title/Abstract]) OR (RCT[Title/Abstract]) OR (controlled clinical trial[Title/Abstract]) OR (controlled clinical trials[Title/Abstract]) OR (random*[Title/Abstract]) OR (control*[Title/Abstract]) OR (blind[Title/Abstract]) OR (blind*[Title/Abstract]) OR (placebo[Title/Abstract]) | 5193582 |
| #11 | #8 OR #9 OR #10 | 5246431 |
| #12 | #3 AND #7 AND #11 | 2414 |
| EMBASE | | |
| #1 | 'metformin'/exp | 79758 |
| #2 | 'biguanide'/exp | 2421 |
| #3 | biguanide*:ab,ti OR metformin:ab,ti OR dimethylbiguanidine:ab,ti OR dimethylguanylguanidine:ab,ti OR glucophage:ab,ti OR biguanide:ab,ti | 44791 |
| #4 | #1 OR #2 OR #3 | 83143 |
| #5 | 'neoplasm'/exp | 5642145 |
| #6 | tumor:ab,ti OR tumo*:ab,ti OR neoplasm:ab,ti OR neoplas*:ab,ti OR cancer:ab,ti OR cancer*:ab,ti OR malignan*:ab,ti OR benign:ab,ti OR carcinoma:ab,ti OR carcinoma*:ab,ti | 5163602 |
| #7 | #5 OR #6 | 6721248 |
| #8 | 'randomized controlled trial (topic)'/exp | 227163 |
| #9 | 'controlled clinical trial (topic)'/exp | 235740 |
| #10 | 'randomized controlled trial':ab,ti OR 'randomized controlled trials':ab,ti OR rct:ab,ti OR 'controlled clinical trial':ab,ti OR 'controlled clinical trials':ab,ti OR random*:ab,ti OR control*:ab,ti OR blind:ab,ti OR blind*:ab,ti OR placebo:ab,ti | 6785887 |
| #11 | #8 OR #9 OR #10 | 6879431 |
| #12 | #4 AND #7 AND #11 | 4623 |
| Cochrane | | |
| #1 | MeSH descriptor: [Metformin] explode all trees | 4487 |
| #2 | MeSH descriptor: [Biguanides] explode all trees | 7238 |
| #3 | ((metformin) or (dimethylbiguanidine) or (dimethylguanylguanidine) or (glucophage) or (biguanide) or (biguanid*)):ti,ab,kw | 12208 |
| #4 | MeSH descriptor: [Randomized Controlled Trials as Topic] explode all trees | 15180 |
| #5 | MeSH descriptor: [Controlled Clinical Trials as Topic] explode all trees | 15334 |
| #6 | ((randomized controlled trial) or (randomized controlled trials) or (RCT) or (controlled clinical trial) or (controlled clinical trials) or (random*) or (control*) or (blind) or (blind*) or (placebo)):ti,ab,kw | 1439361 |
| #7 | MeSH descriptor: [Neoplasms] explode all trees | 88476 |
| #8 | ((tumor) or (tumo*) or (neoplasm) or (neoplas*) or (cancer) or (cancer*) or (malignan*) or (benign) or (carcinoma) or (carcinoma*)):ti,ab,kw | 246493 |
| #9 | #1 OR #2 OR #3 | 14766 |
| #10 | #4 OR #5 OR #6 | 1439380 |
| #11 | #7 OR #8 | 256203 |
| #12 | #9 AND #10 AND #11 | 1382 |
