# Supplementary material for: Efficacy of metformin therapy in patients with cancer: a meta-analysis of 22 randomised controlled trials
Source: BMC Med. 2022 Oct 24;20:402. doi: 10.1186/s12916-022-02599-4 (PMC9594974; doi:10.1186/s12916-022-02599-4)
Supplement: Supplementary file 2 — Additional file 2: Table S1. Population characteristics of included studies. [file 12916_2022_2599_MOESM2_ESM.docx]

**Additional file 2**

**Table S1**: Population characteristics of included studies

| Study | Mean age (years) | | Female (%) | | Mean BMI (kg/m^2^) | |
| --- | --- | --- | --- | --- | --- | --- |
|  | Metformin | Control | Metformin | Control | Metformin | Control |
| Goodwin | 52 | 53 | 99.9 | 99.9 | 27 | 28 |
| Pimentel | 55 | 57 | 100 | 100 | 26.5 | 26.6 |
| Nanni | 57 | 61 | 100 | 100 | / | / |
| Zhao | 57.5 | 56.5 | 100 | 100 | / | / |
| Salah | 48.8 | 49.2 | 100 | 100 | <25 kg/m^2^ 44%  >25 kg/m^2^ 56% | <25 kg/m^2^ 40%  >25 kg/m^2^ 60% |
| Liubota | 57.9 | 58.3 | 100 | 100 | / | / |
| EL-Haggar | 49.78 | 48.84 | 100 | 100 | 29.39 | 30.46 |
| Hamedi | 49.7 | 47.5 | 100 | 100 | / | / |
| Zheng | 53.55 | 52.88 | 100 | 100 | 23.23 | 23.60 |
| Bae-Jump | 65 | 64 | 100 | 100 | <30 kg/m^2^ 49.6%  >30 kg/m^2^ 51.4% | <30 kg/m^2^ 49.8%  >30 kg/m^2^ 50.2% |
| Alghandour | 67 | 69 | 0 | 0 | <30 kg/m^2^ 58.1%  >30 kg/m^2^ 41.9% | <30 kg/m^2^ 80.6%  >30 kg/m^2^ 19.4% |
| Martin | 70 | 69 | 0 | 0 | 27.2 | 26.1 |
| Li | 59.6 | 58.3 | 58.9 | 56.8 | / | / |
| Arrieta | 58.4 | 60.4 | 59.4 | 71.4 | / | / |
| Lee | <65 years 50.6%  >65 years 49.4% | <65 years 56.6%  >65years 43.4% | 11.1 | 19.2 | / | / |
| Marrone | 58 | 64 | 63.1 | 50 | / | / |
| Sayed | 56 | 52 | 20 | 13.3 | / | / |
| Skinner | 63 | 64 | 43 | 40.7 | / | / |
| Tsakiridis | 65.9 | 65.3 | 53.8 | 57.1 | 26.5 | 26.2 |
| Kordes | 64 | 65 | 43.3 | 55.7 | / | / |
| Reni | 64 | 63 | 45.2 | 55.1 | / | / |
| Shorbagy | <60 years 42.5%  >60 years 57.5% | <60 years 47.5%  >60 years 52.5% | 25 | 22.5 | / | / |
