# Supplementary material for: Efficacy of metformin therapy in patients with cancer: a meta-analysis of 22 randomised controlled trials
Source: BMC Med. 2022 Oct 24;20:402. doi: 10.1186/s12916-022-02599-4 (PMC9594974; doi:10.1186/s12916-022-02599-4)
Supplement: Supplementary file 3 — Additional file 3: Fig. S1. Risk of bias summary. Fig. S2. Risk of bias graph for each included study. Fig. S3. Sensitivity analyses for PFS. Fig. S4. Sensitivity analyses for OS. Fig. S5. Meta-regression for OS by maintenance dose. Fig. S6. Funnel plots for PFS and OS. [file 12916_2022_2599_MOESM3_ESM.docx]

**Additional file 3**

**Figure S1**: Risk of bias summary

**Figure S2**: Risk of bias graph for each included study

**Figure S3**: Sensitivity analyses for PFS

**Figure S4**: Sensitivity analyses for OS

**Figure S5**: Meta-regression for OS by maintenance dose

**Figure S6**: Funnel plots for PFS and OS

**Figure S1**: Risk of bias summary: review authors' judgements about each risk of bias item presented as percentages across all included studies


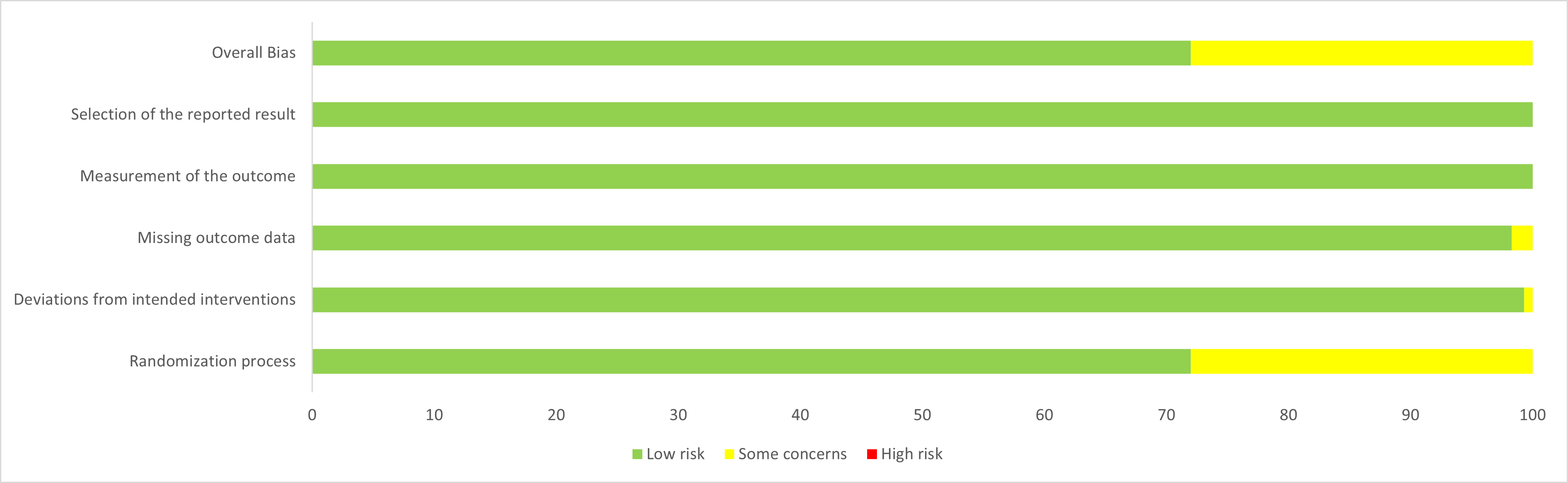


**Figure S2**: Risk of bias graph: review authors' judgements about each risk of bias item for each included study.

| Study | D1 | D2 | D3 | D4 | D5 | Overall |  |  |  |
| --- | --- | --- | --- | --- | --- | --- | --- | --- | --- |
| Salah-2021 | 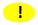 | 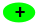 | 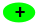 | 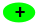 | 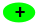 | 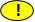 |  | 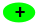 | Low risk |
| Bae-Jump-2020 | 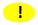 | 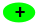 | 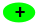 | 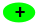 | 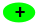 | 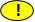 |  | 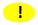 | Some concerns |
| Martin-2021 | 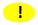 | 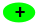 | 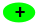 | 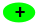 | 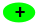 | 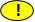 |  | 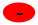 | High risk |
| Alghandour-2021 | 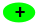 | 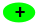 | 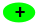 | 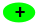 | 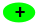 | 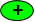 |  |  |  |
| Nanni-2019 | 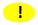 | 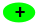 | 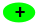 | 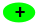 | 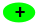 | 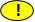 |  | D1 | Randomisation process |
| Pimentel-2019 | 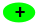 | 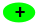 | 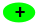 | 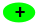 | 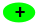 | 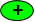 |  | D2 | Deviations from the intended interventions |
| Zhao-2017 | 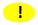 | 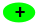 | 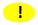 | 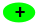 | 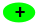 | 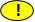 |  | D3 | Missing outcome data |
| EL-Haggar-2016 | 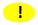 | 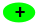 | 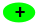 | 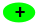 | 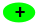 | 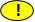 |  | D4 | Measurement of the outcome |
| Liubota-2018 | 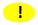 | 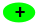 | 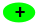 | 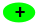 | 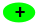 | 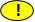 |  | D5 | Selection of the reported result |
| Hamedi-2018 | 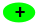 | 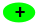 | 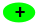 | 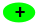 | 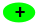 | 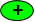 |  |  |  |
| Zheng-2019 | 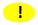 | 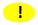 | 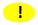 | 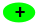 | 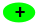 | 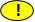 |  |  |  |
| Kordes-2015 | 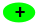 | 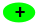 | 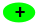 | 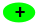 | 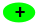 | 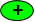 |  |  |  |
| Reni-2016 | 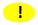 | 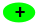 | 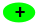 | 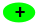 | 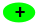 | 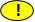 |  |  |  |
| Lee-2021 | 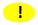 | 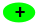 | 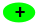 | 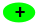 | 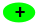 | 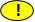 |  |  |  |
| Arrieta-2019 | 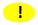 | 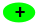 | 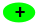 | 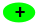 | 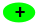 | 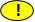 |  |  |  |
| Marrone-2018 | 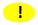 | 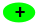 | 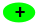 | 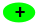 | 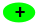 | 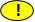 |  |  |  |
| Skinner-2021 |  |  |  |  |  |  |  |  |  |
| Li-2019 |  |  |  |  |  |  |  |  |  |
| Tsakiridis-2021 |  |  |  |  |  |  |  |  |  |
| Sayed-2015 |  |  |  |  |  |  |  |  |  |
| Shorbagy-2020 |  |  |  |  |  |  |  |  |  |
| Goodwin-2022 |  |  |  |  |  |  |  |  |  |

**Figure S3**: Sensitivity analyses for PFS

**Figure S4**: Sensitivity analyses for OS

**Figure S5**: Meta-regression for OS by maintenance dose

**Figure S6**: Funnel plots for PFS and OS
